# Blood Pressure Profiles in Infants With Hypoxic Ischemic Encephalopathy (HIE), Response to Dopamine, and Association With Brain Injury

**DOI:** 10.3389/fped.2020.00512

**Published:** 2020-08-26

**Authors:** Christine Pazandak, Christopher McPherson, Maryam Abubakar, Santina Zanelli, Karen Fairchild, Zachary Vesoulis

**Affiliations:** ^1^Division of Newborn Medicine, Department of Pediatrics, Washington University School of Medicine, Saint Louis, MO, United States; ^2^Department of Pediatrics, University of Virginia, Charlottesville, VA, United States

**Keywords:** neonate, neurology, dopamine, blood pressure, hypoxic ischemic encephalopathy, seizures, brain injury, therapeutic hypothermia

## Abstract

**Objective:** To describe mean arterial blood pressure (MABP), responsiveness to dopamine, and relationship to brain injury in infants with moderate/severe hypoxic-ischemic encephalopathy (HIE) undergoing therapeutic hypothermia (TH). We hypothesized that, when utilized, dopamine would rapidly and effectively increase MABP in treated patients.

**Methods:** Continuous arterial blood pressure measurements were prospectively recorded from infants with moderate/severe HIE undergoing TH in a multi-institutional cohort from 2010 to 2018. Treatment with dopamine was at the discretion of the medical team for hypotension/hypoperfusion. MABP values of treated infants were compared to those obtained at an equivalent time period in control infants receiving TH but not dopamine (24 h after birth). MRI was obtained per unit protocols and included T1/T2/DWI sequences. Injury was classified as no injury/mild injury or moderate/severe injury using a standardized scoring system. Seizures were confirmed with conventional EEG.

**Results:** Eighteen infants were treated with dopamine and were similar to untreated controls (*n* = 36) with the exception of lower cord gas pH (6.92 ± 0.2 vs. 7.07 ± 0.2, *p* < 0.05). Dopamine was initiated at a mean of 24 h after birth. MABP was significantly lower in the dopamine group at the start of therapy (39.9 ± 2.0 vs. 49.1 ± 1.3, *p* < 0.01) and 1 h later (44.3 ± 2.0 vs. 49.8 ± 1.1, *p* < 0.05). However, after 9 h of treatment, dopamine increased the MABP by an average of 9 mmHg and MABP values were similar to untreated controls for the remainder of the observation period. There were no significant differences in rates of seizures, brain injury, or death.

**Conclusion:** Neonates with moderate/severe HIE treated with dopamine during TH had MABP significantly lower than controls. The majority of infants responded to dopamine monotherapy following adequate volume resuscitation. An association between requirement for dopamine and severity of brain injury was not detected.

## Introduction

Hypoxic-ischemic encephalopathy (HIE) secondary to birth asphyxia is a significant cause of neonatal morbidity and mortality, affecting 1-8/1,000 live births in developed countries (1). Throughout the world, HIE is responsible for one-tenth of all disability adjusted life years (2). Although birth asphyxia may be caused by a myriad of perinatal events including umbilical cord accidents, placental abruption, fetal entrapment, and fetal blood loss, the underlying mechanism of injury follows a common pathway (3). In each case, injury arises from an imbalance between oxygen supply and demand leading to a clinical syndrome known as HIE. The clinical presentation of HIE is broad with signs and symptoms ranging from mild encephalopathy to multi-organ system failure, autonomic instability, absence of primitive reflexes, seizures, and death (4, 5).

Therapeutic hypothermia (TH) became the standard of care for moderate to severe HIE after multiple randomized control trials demonstrated a reduction in the combined outcome of death or moderate-severe disability (6–9). TH exerts a neuroprotective effect by leveraging kinetic properties of temperature-dependent enzymatic reactions within the body, thus slowing the rate of oxygen consumption and demand for ATP (10, 11) and preventing secondary energy failure. Although TH improves outcomes in patients with HIE, brain injury and adverse neurodevelopmental outcomes are still common. Adjunctive interventions in targeted populations are needed to further reduce morbidity and mortality.

One potential adjunctive target for intervention is optimizing cerebral perfusion. For infants with HIE, both the underlying disease process *and* the treatment of HIE have the potential to negatively impact the hemodynamic status of the infant, and this is frequently manifested as hypotension (12). Studies of the optimal approach to hemodynamic support in neonates are lacking in general, and existing literature is primarily focused on premature infants. While the goal of therapeutic hypothermia is to improve metabolic mismatch, it may not be enough in many cases, prompting the use of adjunctive measures.

The response to one of the most common interventions, dopamine, in a population of HIE infants has only been reported in a single small study (12) leaving clinicians with limited data for guidance. Dopamine, a sympathomimetic amine that acts through direct stimulation of alpha-, beta-, and dopaminergic receptors and indirect stimulation of dopamine2 receptor causing the release of norepinephrine, remains the most commonly used medication to treat neonatal hypotension (13). Although there is a wealth of observational and randomized studies evaluating the efficacy of dopamine for the treatment of hypotension in the preterm population (14–16), there are limited neonatal data (17) and inconsistent animal data (18–21) regarding the efficacy of dopamine in asphyxiated infants.

In this study, we evaluate the blood pressure response to dopamine in infants diagnosed with hypotension/hypoperfusion after moderate/severe HIE treated with TH. In addition, we explore the relationship between the requirement for dopamine treatment and brain injury. We hypothesized that a subset of infants with HIE would require treatment with dopamine for low mean arterial blood pressure (MABP) and that dopamine would rapidly and effectively increase MABP in treated patients.

## Materials and Methods

### Study Design and Patient Population

This retrospective, multi-center case-control study was conducted in the neonatal intensive care units (NICU) of St. Louis Children's Hospital (SLCH) and University of Virginia Children's Hospital (UVa) from 2010 to 2018. Both centers are Level IV units serving patients from urban, suburban, and rural populations. Infants were included in the study if they underwent TH for the treatment of neonatal encephalopathy as determined by a modified Sarnat exam (22) and had an intra-arterial catheter placed for invasive blood pressure monitoring. Treatment with dopamine was at the discretion of the medical team and given for signs or symptoms of hypotension/hypoperfusion.

Cases were determined by exposure to dopamine and were matched to controls 1:2 by gestational age and gender. Infants in the control group were otherwise identical except for exposure to dopamine. Infants were excluded if there was a known congenital or genetic anomaly at the time of birth. To prevent confounding, infants were excluded if they were exposed to other inotropes or vasopressors (i.e., norepinephrine, epinephrine, and milrinone). Infants were also excluded if blood pressure data were not available at the time of dopamine initiation or the comparable period in control infants.

### Institutional Practices

At SLCH and UVa, universal arterial and venous cord gas screening is performed at all inborn deliveries. At both institutions, standardized encephalopathy exams are performed within the first 6 h of life for infants ≥ 34 weeks who are at risk for neonatal encephalopathy (defined as a pH < 7.10 or a base deficit <12 at SLCH and pH < 7.0 or base deficit <16 at UVa). TH treatment is initiated within 6 h of birth for all infants with qualifying exams.

The standard whole-body TH protocol includes 72 h of servo-controlled hypothermia at 33.5°C followed by 12–24 h of rewarming to 36.5°C. At SLCH, conventional EEG is performed for a minimum of 24 h with at least one non-sedated MRI completed within 14 days of life; at UVa, conventional EEG is performed for the entire duration of cooling and rewarming and non-sedated MRI is completed immediately after rewarming (days 3-5) or after day 10 (if unable to obtain an early MRI).

To prevent shivering and maximize the benefits of TH, sedation is provided to infants at both institutions per protocol. At SLCH, morphine is utilized for sedation with an initial bolus (50 mcg/kg) followed by a continuous infusion (10 mcg/kg/h for 12 h and then decreased to 5 mcg/kg/h) for the duration of cooling. At UVa, continuous infusion of either fentanyl 0.5 mcg/kg/h or dexmedetomidine 0.2 mcg/kg/h (from 2014 to 2016) are used during cooling. At both centers, increased infusions or bolus doses of sedatives were given as needed for agitation.

Dopamine is the first-line medication used to treat hypotension/hypoperfusion in an infant with HIE undergoing TH at both institutions and is often given in conjunction with or following a normal saline bolus. In this study, dopamine was initiated and escalated at the discretion of the clinical treatment team. If the infant did not respond to dopamine and a second agent was needed, blood pressure data were not analyzed during that time period.

### Data Collection

#### Clinical Factors

Clinical characteristics were obtained from the electronic medical record including maternal age, antenatal magnesium exposure, mode of delivery, gestational age, worst degree of encephalopathy in the first 6 h, birth weight, Apgar scores (1, 5, and 10 min), cord gas pH, intubation and mechanical ventilation in the first 96 h, and inborn/outborn status.

#### EEG Monitoring

Both institutions utilize conventional video EEG to monitor for the presence of seizures. Electrodes are placed using the standard International 10–20 system, modified for neonates and EEG monitoring is initiated as soon as possible after cooling starts. Seizures were defined using the aCNS consensus definition, namely a rhythmic electrographic event which is of sudden onset, repetitive and evolving, with a duration of at least 10 s (23).

#### Medication Data

A comprehensive review of the medication administration record was performed. The following information was collected for dopamine, morphine, fentanyl, dexmedetomidine, midazolam, and vecuronium: cumulative bolus dose (mg/kg or mcg/kg), infusion start date/time, infusion stop date/time, initial infusion dose (mcg/kg/min, mcg/kg/h, or mg/kg/h), maximum infusion dose, and cumulative infusion dose. Additionally, the dose (mcg/kg/min) of dopamine at 1, 3, 6, 9, 12, 18, 24, 36, 48, and 72 h after dopamine initiation was collected. The number of normal saline boluses in addition to dates/times and dose (mL/kg) were collected. The date/time, dose (mg/kg), and frequency of hydrocortisone administration were collected. Exposure to anti-epileptic medications (dates/times and doses in mg/kg), including phenobarbital, fosphenytoin, midazolam, and levetiracetam, were also recorded. Initiation of maintenance anti-epileptic dosing was noted.

#### Transfusion Data

The number, dates, and times of packed red blood cell (pRBC), platelet, and fresh frozen plasma (FFP) transfusions were extracted from the transfusion record.

#### Neuroimaging

After completion of TH, all surviving neonates underwent non-contrast, non-sedated MRI examination following institutional MRI guidelines. Brain MRI imaging was performed with either a Siemens 1.5-T Avanto/Aera or 3.0-T Trio/Skyra/Prisma (Siemens Medical, Erlangen, Germany) and included T1/T2/DWI sequences. MRI images were reviewed by pediatric neuroradiologists blinded to blood pressure outcomes and scored using a standardized HIE scoring system (24). Briefly, this system examines five regions of the brain (cortex, white matter, cerebellum, subcortical gray matter and brain stem) assigning points based on increasing severity of injury across each of the three sequences. Using neurodevelopmental outcome data for validation, injury can be classified in four categories: no injury (score=0), mild injury (score=1–11), moderate injury (score=12–32), and severe injury (score=33–138).

Given the small sample size, injury was classified as a binary variable using the categorical output of the scoring system; normal/mild injury vs. moderate/severe injury. If two scans were obtained in the same infant, the scan with the worst injury was recorded.

### Blood Pressure Analyses

Blood pressure data was obtained via umbilical arterial lines that were placed at the discretion of the clinical treatment team. Per standard clinical practice, umbilical arterial lines are placed in a manner so that the tip of the catheter lies between the sixth and eighth thoracic or the third and fourth lumbar vertebrae on radiograph. Continuous, invasive arterial blood pressure measurements were recorded using a pressure transducer which interfaces with the umbilical arterial catheter and patient monitor (SLCH: IntelliVue MP70 or MX800, Philips Medical, Andover, MA and UVa: GE CARESCAPE B850, GE Medical System, Chicago, IL).

MABP data were prospectively collected with a sampling rate of 0.5 Hz and archived in a database (BedMasterEx, ExcelMedical, Jupiter, FL). The files were then converted to a MATLAB (The Math Works, Natick, MA) matrix for analysis. As blood pressure is known to increase in a linear fashion following birth (25, 26), comparison of average MABP at equivalent time points in the case and control groups was essential. For infants in the case group, T_0_ was defined at the time of dopamine initiation. Empiric evaluation of the case group revealed that dopamine was consistently started around 24 h of life. For infants in the control group, T_0_ was defined as the point 24 h following birth.

From T_0_, we calculated the average MABP over a 10-min interval centered at the time points of 1, 3, 6, 9, 12, 18, 24, 36, 48, and 72 h. In the instance where a MABP value was not available for an infant at a specific time point, an average MABP was calculated from the remaining infants.

### Statistical Analyses

Clinical factors were compared between cases and controls by using Chi-square or Fisher's exact test for categorical variables and Mann–Whitney *U*-test for continuous variables (IBM, Statistics SPSS, 25). Statistical significance was accepted as *p* < 0.05.

## Results

### Cohort Characteristics

During the study period, 98 infants were diagnosed with moderate-severe encephalopathy and had arterial lines placed at SLCH. Of those 98 infants, 30 were excluded due to poor data quality, exposure to other inotropes, or congenital anomalies. The remaining 68 patients included seven infants treated only with dopamine. After matching controls in a 2:1 fashion, 16 controls, and seven cases remained. At UVa, 78 infants were diagnosed with moderate-severe encephalopathy and had arterial lines placed during the study period. Of those 78 infants, 28 were excluded due to the same exclusions listed above. The remaining 60 patients included 11 patients treated only with dopamine. After matching controls in a 2:1 fashion, 20 controls and 11 cases remained in the study.

The final cohort consisted of 54 infants with 18 cases matched to 36 controls. Many of the clinical and demographic characteristics between the cases and controls were statistically similar (including severity of encephalopathy at start of TH), but the two groups differed with respect to their cord gases with dopamine-treated infants demonstrating lower pH indicating more severe asphyxia (6.9 ± 0.2 vs. 7.1 ± 0.2, *p* < 0.01, Table 1).

**Table 1:** Clinical and demographic characteristics of study cohort.

	**Dopamine treatment (cases), *n* = 18**	**No dopamine treatment (controls), *n* = 36**	***P*-value**
Gestational age at birth, mean ± SD, weeks	39 ± 1.7	38 ± 1.5	0.70
Male sex, *n* (%)	11 (61)	21 (58)	0.85
Mode of delivery			
Vaginal, *n* (%)	6 (33)	18 (50)	0.25
C-section, *n* (%)	12 (66)	18 (50)	
Birth weight, mean ± SD, grams	3257 ± 541.3	3333 ± 498.9	0.20
Race			
Caucasian, *n* (%)	12 (67)	28 (78)	0.38
African American, *n* (%)	6 (33)	6 (17)	0.17
Asian, *n* (%)	0	1 (3)	1.00
Native American, *n* (%)	0	1 (3)	1.00
Maternal age, mean ± SD, years	27 ± 6.9	28 ± 5.4	0.62
Clinically diagnosed chorioamnionitis, *n* (%)	2 (17)^a^	2 (6)	0.26
Antenatal Magnesium Exposure, *n* (%)	1 (6)	2 (6)	1.00
Apgar scores, median (interquartile range) (1, 5, and 10 min)	1 (2.3)	2 (2)	0.14
	3 (2)	4 (4)	0.24
	4 (4)^b^	5 (3)^b^	0.82
Cord pH, mean ± SD	6.92 ± 0.2	7.07 ± 0.2	<0.01^c^
Intubated within 96 h of life, n (%)	16 (89)	22 (61)	0.04^c^
Inborn, n (%)	9 (50)	19 (52)	0.85
Worst severity of encephalopathy in first 6 h, *n* (%)			
Mild	1 (5)	6 (17)	0.43
Moderate	14 (78)	27 (75)	
Severe	3 (17)	3 (8)	

a *Clinically diagnosed chorioamnionitis has 6 missing data points in the dopamine treated group*,

b *1 infant did not have 1-min Apgar score and 3 infants did not have assigned 10-min Apgar scores*,

c *denotes significance at the p < 0.05 level*.

The cases in this cohort received more hydrocortisone and cumulative fentanyl, but similar amounts of morphine compared to controls (each institution has different sedation protocols). There were no other differences in pharmacologic interventions (Table 2). Although there was a trend toward increased seizures confirmed by conventional EEG and a greater incidence of moderate to severe brain injury in the dopamine-treated group, these differences were not statistically significant. Additionally, there was no significant difference in the rate of death between the two groups (Table 3).

**Table 2:** Summary of medications received with 96 h of life.

	**Dopamine treatment (cases), *n* = 18**	**No dopamine treatment (controls), *n* = 36**	***P*-value**
Morphine, *n* (%)	7 (39)	16 (44)	0.70
Morphine, cumulative infusion dose, mcg/kg/h, mean ± SD	541.1 ± 245	642.3 ± 337	0.45
Fentanyl, *n* (%)	12 (67)	20 (56)	0.43
Fentanyl, cumulative infusion dose, mcg/kg/h, mean ± SD	102.5 ± 32	63.1 ± 24.9	<0.01^a^
Dexmedetomidine, n (%)	1 (6)	0	0.33
Midazolam, *n* (%)	4 (22)	4 (11)	0.42
Vecuronium, *n* (%)	0	0	
Hydrocortisone, *n* (%)	3 (17)	0	0.03^a^
Phenobarbital, *n* (%)	7 (39)	9 (25)	0.29
Phenobarbital maintenance, *n* (%)	3 (17)	4 (11)	1.00
Fosphenytoin, *n* (%)	1 (6)	0	0.33
Levetiracetam, *n* (%)	1 (6)	2 (6)	1.00
Levetiracetam maintenance, *n* (%)	1 (6)	2 (6)	1.00

a *denotes significance at the p < 0.05 level*.

**Table 3:** Neonatal outcomes.

	**Dopamine treatment (cases), *n* = 18**	**No dopamine treatment (controls), *n* = 36**	***P*-value**
Seizures, *n* (%)	7 (39)	6 (17)	0.07
MRI injury, moderate to severe, *n* (%)	5 (28)	6 (17)	0.30
Death, *n* (%)	2 (11)	0	0.12

a *denotes significance at the p < 0.05 level*.

### Data Quality

The average age at the start of the recording was 5.6 ± 3.5 h and the average length of each recording was 66.4 ± 3.5 h. The average duration of dopamine therapy was 66.1 ± 38.8 h. One dopamine-treated infant had one missing blood pressure reading at 96 h of life. The control group had 1, 3, 5, and 13 missing MABP measures at 48, 60, 72, and 96 h of life, respectively. Missing data can be attributed to discontinuation of the umbilical arterial line by the clinical treatment team prior to the end of the study period.

### Volume Resuscitation

There was no statistically significant difference between dopamine-exposed and control infants in the frequency or volume of normal saline boluses given for hypotension/hypoperfusion. Similarly, there was no difference in the frequency or volume of pRBC transfusion for the treatment of anemia. In contrast, the dopamine-exposed infants had a greater frequency of exposure to platelet and FFP transfusions compared to controls (*p* < 0.05, Table 4).

**Table 4:** Summary of volume and blood product resuscitation received within 96 h of life.

	**Dopamine treatment (cases), *n* = 18**	**No dopamine treatment (controls), *n* = 36**	***P*-value**
NS, *n* (%)	15 (83)	24 (67)	0.33
NS, average total cumulative volume, mls/kg, mean ± SD	32 ± 16	25 ± 18	0.19
pRBC transfusion, *n* (%)	5 (28)	3 (8)	0.10
pRBC, average total cumulative volume, mls/kg, mean ± SD	25 ± 8	15 ± 0	0.25
Plt transfusion, *n* (%)	5 (28)	2 (6)	0.03^a^
Plt, average total cumulative volume, mls/kg, mean ± SD	32 ± 16	23 ± 11	0.57
FFP transfusion, *n* (%)	8 (44)	7 (19)	0.05^a^
FFP, average total cumulative volume, mls/kg, mean ± SD	46 ± 38	18 ± 9	0.09

*NS, Normal saline; pRBC, packed red blood cell; FFP, fresh frozen plasma*,

a *denotes significance at the p < 0.05 level*.

### Blood Pressure Outcomes

**Figure 1:**
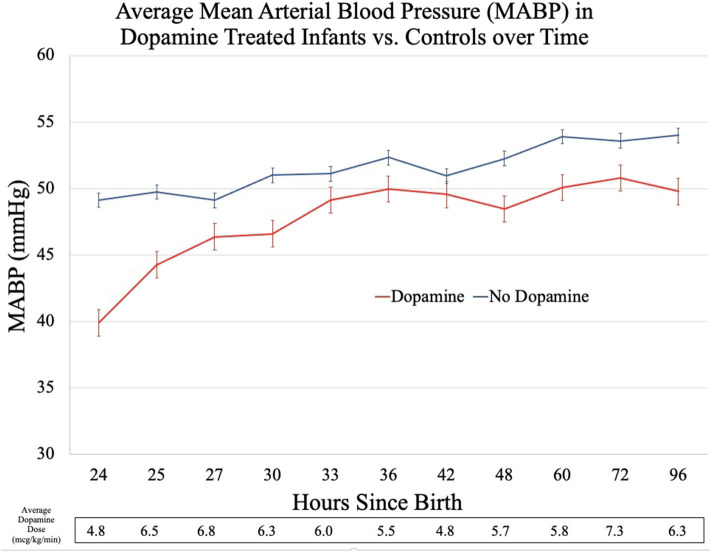
Hemodynamic changes: MABP in dopamine treated infants vs. controls.

Compared to controls, the dopamine-exposed infants had a significantly lower mean MABP at T_0_, a difference of ~9 mmHg (*p* < 0.01, Figure 1). In the 1-h period following T_0_, the mean MABP increased by an average of 4 mmHg for the dopamine-exposed infants, compared to no change in the control infants. The average MABP in the dopamine-exposed infants continued to increase over time and matched the starting (T_0_) average MABP of the control infants at the 9th h of dopamine treatment.

In addition to the absolute differences in average MABP, the two groups of infants had markedly different MABP trajectories. The dopamine-treated infants had irregular changes in MABP with time, increasing more rapidly after dopamine initiation before appearing to plateau at 49–50 mmHg. This stands in contrast to the control infants who continued to have a smooth, uninterrupted increase in MABP as postnatal age increased.

## Discussion

This study provides a comprehensive evaluation of blood pressure changes occurring in infants with neonatal encephalopathy undergoing TH with reliable data from continuous, invasive blood pressure monitoring. We found that infants with neonatal encephalopathy undergoing TH and treated with dopamine received more blood products and generally responded to dopamine treatment, although a small subset required exogenous adrenal replacement. Additionally, although there was a trend toward a greater number of seizures and increased incidence of moderate to severe brain injury for dopamine-treated infants, it did not meet statistical significance. There was no difference in mortality between the groups.

Infants requiring dopamine treatment in our cohort had similar demographic and clinical characteristics compared to controls but were more severely asphyxiated as demonstrated by the lower pH in the cord blood. Despite this difference, those more severely asphyxiated received a similar frequency and volume of fluid resuscitation compared to the control group, a finding in line with previously published data (12). However, we found that dopamine-treated infants required more platelet and fresh frozen plasma transfusions compared to controls. This is consistent with previous literature indicating that encephalopathy and TH are associated with coagulation abnormalities such as thrombocytopenia and disseminated intravascular coagulation (27–29). In fact, Sweetman et al. found that coagulation profiles during the first few days of life could predict early clinical outcomes, such as the need for therapeutic hypothermia and severity of encephalopathy (27). As all infants in this study were treated with TH, the association between dopamine treatment and blood product administration is most likely the result of a greater degree of asphyxia (as indicated by the lower pH in the cord blood) and subsequent coagulopathy.

This study is the first to report the blood pressure response to dopamine of infants diagnosed with HIE undergoing TH and is strengthened by the availability of concurrent, continuous, full EEG monitoring. Only one previous study, published by DiSessa et al., describes the cardiovascular effects of dopamine in severely asphyxiated neonates (17). However, this study was performed before the advent of TH, only included 14 total infants, and randomized infants to a prophylactic treatment of dopamine with a maximum dose of 2.5 mcg/kg/min. Similar to DiSessa, we found that dopamine generally increased blood pressure in asphyxiated infants after adequate volume resuscitation; however, response was more rapid in the previous study. The more granular data during the first 96 h of life in infants in the current cohort compared to DiSessa's average of 4 hourly time points pre- and post-dopamine administration allowed us to detect delayed normalization of blood pressure in neonates with moderate/severe HIE undergoing TH requiring dopamine compared to untreated controls.

We noted a clinically important, although not statistically significant, difference in seizure incidence between dopamine-exposed and control infants. Seizures are a common sequela of an initial ischemic injury, portend a worse outcome (30–32), and represent ongoing cerebral injury, potentially partially due to inadequate cerebral perfusion. While cerebral perfusion was not directly measured in this study, it is concerning that seizures tended to accompany other signs of impaired systemic perfusion. Indeed, instability of blood pressure and the severity of HIE are likely to be intrinsically linked, causing a pathologic synergism which exposes the brain to further injury and manifests as seizures. Fortunately, this observation hints at the possibility of intervention; if an impaired perfusion state can be detected earlier and addressed, additional seizure-related injury might be partially avoided. The etiology and evolution of brain injury in infants with HIE is complex and results from the interaction between the initial perinatal insult and later NICU challenges. Although it is not possible to differentiate the exact degree of risk posed by postnatal events, the mechanism of additional injury is plausible and addressable; identifying early markers of inadequate cerebral perfusion and associated intervention trials are urgently needed.

By design, infants who did not respond to dopamine were excluded from this study. By using a group of control infants who received no inotropes and a case group who received a single agent, this comparison could be made with the fewest confounders and have the greatest generalizability for typical treatment of the typical infant. While dopamine non-responders deserve investigation, this line of investigation would be confounded by the small number of infants in this group and the broad diversity of second-line treatments (e.g., milrinone, dobutamine, norepinephrine, ECMO) which have very different mechanisms of action.

We did not find significant differences in frequency of brain injury on MRI between the dopamine-exposed and control infants. This result was surprising, as there is considerable evidence that hypotension/hypoperfusion states are associated with impaired cerebral autoregulation, a known risk factor for brain injury (33–36). Disruption of autoregulation allows for pathologic perfusion states to occur, in both directions, and potentiates ischemia-reperfusion injury. While the results of this study suggest a link between asphyxia and altered hemodynamics, MABP measures alone likely provide an inadequate view of cerebral perfusion.

There are several potential reasons for the null finding in this study. First, it is possible that need for dopamine is not linked with additional risk for brain injury and that injury is merely the result of the original hypoxic-ischemic event. More likely is that MABP is a crude measure of perfusion; MABP is captured via pressure catheter at the level of a large vessel and may not reflect the complex of factors which govern perfusion in more distal vessels. Indeed, the entire concept of normal or adequate blood pressure threshold in neonates remains in question (25). Of note, the mean MABP measurements of the dopamine-exposed infants in this study was still above the widely accepted threshold of MABP > gestational age in weeks (37).

The methodology of this study was practical in nature, utilizing readily available measurements of MABP. These results suggest that the interaction between systemic and cerebral blood flow requires interrogation at the cerebrovascular level. The use of transcranial Doppler ultrasound to measure cerebral blood flow velocity and evaluate the resistive index might provide one such alternative. While this approach directly measures cerebral blood flow velocity, it is not practical for continuous monitoring due to tissue heating (38, 39) and may interfere with other monitoring devices such as EEG electrodes, a significant disadvantage compared to MABP which has no such challenge. Near-infrared spectroscopy (NIRS) monitors are non-invasive optical devices capable of measuring cerebral saturation, a proxy for cerebral blood flow, and could be useful in assessment of cerebral autoregulation and adequacy of cerebral perfusion. Although NIRS devices are commercially available, they are not yet universally applied for clinical purposes. Similar challenges exist when trying to find adequate exposed scalp to apply a NIRS probe when concurrent EEG monitoring is being performed. Despite this potential challenge, both methods offer valuable additional insight into cerebral blood flow adequacy after hypoxia and should be included in future studies.

Additional limitations to this study include small sample size, which could have limited our power to detect clinically significant differences in seizures or brain injury, and minor practice variations between the two centers. For example, fentanyl and morphine may have different hemodynamic effects influencing the response to dopamine between the two centers. In addition, differences in timing and interpretation of MR imaging limit the external validity of these findings. It is possible that the differences in radiographic brain injury were sufficiently subtle that they could not be captured when combining infants into normal/mild or moderate/severe categories. Finally, some scans were performed during the time frame where pseudonormalization (40) may have impaired the ability to detect injury, dampening the overall difference between groups.

Considering these limitations, this study highlights the limitations of current clinical practice, namely that the use of MABP and its response to hemodynamic support is inadequate to judge the status of cerebral perfusion and is not helpful for prediction of which infants will have brain injury or seizures. This underscores the urgent need for development of more specific therapeutic endpoints in this population. Future studies should include concurrent measures of cerebral autoregulation (measured using Doppler ultrasound and/or NIRS) and consistent prospective imaging to more fully establish the link between hypotension/hypoperfusion, impaired cerebral blood flow, and risk of brain injury in HIE.

## Conclusion

For infants with hypoxic-ischemic encephalopathy (HIE), both the underlying disease process and therapeutic hypothermia (TH) have the potential to negatively impact the hemodynamic status, and this is frequently manifested as hypotension/hypoperfusion. Dopamine generally increases blood pressure in patients with moderate/severe HIE undergoing TH and facilitates sustained MABP similar to untreated controls during cooling and rewarming. Future studies should include concurrent measures of cerebral autoregulation and consistent prospective imaging to more fully establish the link between hypotension/hypoperfusion, impaired cerebral blood flow, and risk of brain injury in HIE.

## Data Availability Statement

The datasets generated for this study are available on request to the corresponding author.

## Ethics Statement

The study protocol was approved by the Institution Review Boards of Washington University and University of Virginia. The IRB of Washington University in St Louis waived the requirement for written informed consent from the parents/guardians of the participants.

## Author Contributions

Specifically, CP participated in the following aspects of the study: initial concept, study design, data collection, data analysis, data interpretation, draft of first manuscript, and final edits after receiving feedback from other authors. CM participated in study design and data interpretation in addition to providing critical feedback of manuscript drafts. MA, SZ, and KF participated in data collection and data interpretation in addition to providing critical feedback of manuscript drafts. ZV participated in concept, study design, data interpretation, and provided critical review of manuscript drafts. All authors contributed to drafting or revising of the paper for important intellectual content and approve of the final submitted version.

## Conflict of Interest

The authors declare that the research was conducted in the absence of any commercial or financial relationships that could be construed as a potential conflict of interest.
